# Supplementary material for: A Likelihood Approach for Real-Time Calibration of Stochastic Compartmental Epidemic Models
Source: PLoS Comput Biol. 2017 Jan 17;13(1):e1005257. doi: 10.1371/journal.pcbi.1005257 (PMC5240920; doi:10.1371/journal.pcbi.1005257)
Supplement: S1 File — (TAR.GZ) [file pcbi.1005257.s014.tar.gz › HSPH_Online-SI-Revision/PF08/n100-extreme/n100-extreme_PF2_summary2.pdf]

peak −8	3w spec	3w cum	5w spec	5w cum	end	3w spec LOG	3w cum LOG	5w spec LOG	5w cum LOG	end LOG
mean	93.7	96.4	81.4	76.8	17.7	0.5	0.4	0.8	0.6	0.1
median	63.8	54.9	80.7	72.7	18.2	0.5	0.3	0.8	0.6	0.1
quantile .1	54.4	45.1	65.9	59.3	10.3	0.3	0.3	0.5	0.4	0.
quantile .9	131.	156.2	86.6	79.3	22.2	0.6	0.5	0.9	0.4	0.1
14	19	8	8	20						

peak −4	3w spec	3w cum	5w spec	5w cum	end	3w spec LOG	3w cum LOG	5w spec LOG	5w cum LOG	end LOG
mean	47.	45.	31.2	41.1	9.4	0.3	0.3	0.2	0.3	0.
median	48.6	46.2	27.	40.8	7.1	0.3	0.3	0.1	0.2	0.
quantile .1	15.2	14.7	8.5	12.6	2.	0.1	0.1	0.	0.1	0.
quantile .9	74.7	72.1	58.1	67.	18.7	0.6	0.6	0.4	0.1	0.1
2	2	1	2	5						

peak	3w spec	3w cum	5w spec	5w cum	end	3w spec LOG	3w cum LOG	5w spec LOG	5w cum LOG	end LOG
mean	31.7	17.5	128.8	19.6	48.	0.1	0.1	0.3	0.1	0.2
median	20.6	12.2	55.8	17.7	35.9	0.1	0.1	0.2	0.1	0.1
quantile .1	1.5	0.7	10.	2.4	11.7	0.	0.	0.	0.	0.
quantile .9	69.9	40.4	378.6	37.3	107.9	0.2	0.2	0.7	0.	0.3
5	3	0	1	0						

peak +4	3w spec	3w cum	5w spec	5w cum	end	3w spec LOG	3w cum LOG	5w spec LOG	5w cum LOG	end LOG
mean	355.6	192.3	957.8	244.6	348.8	0.5	0.4	0.7	0.4	0.5
median	136.9	87.5	269.6	111.3	184.3	0.4	0.3	0.6	0.3	0.5
quantile .1	32.4	19.6	48.6	24.5	38.6	0.1	0.1	0.2	0.1	0.1
quantile .9	1031.6	544.7	2306.2	662.4	881.3	1.1	0.8	1.4	0.1	1.
0	0	0	0	0						

peak +8	3w spec	3w cum	5w spec	5w cum	end	3w spec LOG	3w cum LOG	5w spec LOG	5w cum LOG	end LOG
mean	2259.1	1284.5	4049.7	1542.3	1957.4	0.8	0.7	1.	0.8	0.8
median	398.	322.4	623.5	371.6	484.4	0.7	0.6	0.9	0.7	0.8
quantile .1	66.8	56.1	100.2	63.3	73.6	0.2	0.2	0.3	0.2	0.2
quantile .9	3833.8	3018.7	9072.1	3590.4	4519.3	1.6	1.5	2.	0.2	1.7
0	0	0	0	0	{}	{}	{}	{}	{}	{}
